# Differing Evolutionary Histories of the ACTN3*R577X Polymorphism among the Major Human Geographic Groups

**DOI:** 10.1371/journal.pone.0115449

**Published:** 2015-02-23

**Authors:** Carlos Eduardo G. Amorim, Victor Acuña-Alonzo, Francisco M. Salzano, Maria Cátira Bortolini, Tábita Hünemeier

**Affiliations:** 1 Departamento de Genética, Instituto de Biociências, Universidade Federal do Rio Grande do Sul, Porto Alegre, Rio Grande do Sul 91501-970, Brazil; 2 CAPES Foundation, Ministry of Education of Brazil, Brasília, 70040-020, Brazil; 3 Unit of Molecular Biology and Genomic Medicine, Instituto Nacional de Ciencias Médicas y Nutrición Salvador Zubirán, Universidad Nacional Autónoma de México, Mexico City 14000, Mexico; Universitat Pompeu Fabra, SPAIN

## Abstract

It has been proposed that the functional ACTN3*R577X polymorphism might have evolved due to selection in Eurasian human populations. To test this possibility we surveyed all available population-based data for this polymorphism and performed a comprehensive evolutionary analysis of its genetic diversity, in order to assess the action of adaptive and random mechanisms on its variation across human geographical distribution. The derived *577X* allele increases in frequency with distance from Africa, reaching the highest frequencies on the American continent. Positive selection, detected by an extended haplotype homozygosisty test, was consistent only with the Eurasian data, but simulations with neutral models could not fully explain the results found in the American continent. It is possible that particularities of Native American population structure could be responsible for the observed allele frequencies, which would have resulted from a complex interaction between selective and random factors.

## Introduction

The human *ACTN3* (α-actinin skeletal muscle isoform 3) gene is located in the long arm of chromosome 11 and encodes an α-actinin binding protein expressed solely in fast twitching skeletal muscle fibers [1]. In polymorphism rs1815739, a C>T transition converts arginine to a premature stop-codon at residue 557 of the ACTN3 protein [2].

Evidence suggests that this polymorphism may affect muscle performance [3] and the derived *577X* allele has been found to be under-represented in sprint/power athletes [4]. While it is believed that ACTN3 is required for optimal muscle performance at high velocity, it is still uncertain if the derived allele could improve endurance performance [3]. Experiments with knock-out mice demonstrated that the lack of ACTN3 in muscle cells results in a significant shift in the metabolic pathways of the lactate-based fast fibers, leading to a slower but more efficient aerobic pathway, normally associated with slow muscle fibers [5]. The lack of this protein is then compensated for by an ACTN2 up-regulation [1].

These data suggest that the high frequency of the *557X* allele in some human populations could have had resulted from selective pressures for increased metabolic efficiency, possibly enhancing the capability for endurance running. This could have favored the emergence of novel kinds of hunting—such as persistence hunting—possibly contributing to range expansions [6]. Subsequently, it was hypothesized that this gene could have evolved under balancing selection earlier in *Homo* history [7] and that more recently it could have been subjected to positive selection in European and Asian populations [5], but not in Africa [8].

Despite the intricate scenario proposed, there have been only a few attempts to analyze it from an evolutionary perspective (for instance, Friedlander et al. [9]). Apart from the Fridlander et al. [9] study, the focus has been exclusively on the association of muscle metabolism and athletes’ performance (Alfred et al. [3] and references therein).

In this study, we asked how human evolutionary history has led to the genetic variation observed at the human *ACTN3*, especially for Native Americans. In order to answer this question, we gathered all available population-based rs1815739 polymorphism data. This created a databank of over 2800 genotyped individuals distributed in 121 autochthonous populations worldwide, the most complete analysis to date of variability of the so-called “athlete gene”. We then used the Extended Haplotype Homozygosity test, estimated the age of emergence of the derived allele, devised five alternative models for the peopling of America to test how well they fit the empirical data for rs1815739, and checked the genotypes for this marker in archaic humans.

## Methods

### Data Source and Populations

Data were first obtained from three public databanks: the 1000 Genomes Project [10], HapMap [11], and the Human Genetic Diversity Panel [12]. Then an extensive literature review retrieved three articles [8, 13, 14] with rs1815739 genotypes from healthy human individuals using random population samples. Our analyses do not include association studies, since these samples were not taken at random. These sources provided an initial set of 3989 genotypes distributed in 150 population samples. From these samples, we eliminated populations with substantial known recent admixture. When individual information on recent admixture was available, we also removed these recent migrants from the data set. Since some of the remaining population samples overlapped in the different reports, only the largest sample size across studies was included. In some cases, the data sources included a couple of populations belonging to the same ethnic group but sampled from two distinct geographic regions (as is the case of Maya and Zapotec in Reich et al. [14]). In those cases, we merged populations with the same name that were separated in the original study. This procedure left us with 2806 genotypes for 121 populations worldwide. Please refer to the primary publications for further information on samples and populations.

Data processing and handling were performed with PGDSpider [15] and with in-house built bash scripts.

### ACTN3*577X Allele Age

To estimate the age of emergence of the derived allele, we used the equation *E*(*t*1) = [−2*p*/(1−*p*)]ln(*p*), where *E*(*t*1) is its expected age in units of 2N generations and *p* is the derived allele frequency [16]. We assumed a generation time of 25 years and *N* = 6000, which is often regarded as a minimum estimate for the effective population size of modern humans during the period before recent growth. Since this formula assumes neutral evolution for the locus under investigation, we used the average derived allele frequency in all pooled selected African populations in the formula, since there is no signal of selection for this continent [8] and no significant population structure for this locus (p = 0.33431; calculated with Arlequin 3.5.1.3 [17]).

### Positive Selection Inference

Additional information on the flanking region of rs1815739 was available [14] for three major human continental groups: 384 individuals from Central and East Asia, 162 from Europe, and 260 from the Americas. This information includes the genotypes for 30 SNPs (rs4930359, rs905770, rs2282529, rs7947391, rs7925108, rs2511224, rs2305535, rs11227501, rs7951189, rs2298806, rs11227516, rs3816492, rs3867132, rs490998, rs2275998, rs540874, rs560556, rs498045, rs556759, rs519380, rs624561, rs10791889, rs3782079, rs664297, rs4930390, rs569818, rs11601241, rs7948839, rs2167457, and rs7119426) located from ∼187 kb upstream to ∼322 kb downstream of rs1815739, therefore spanning circa 510 kb. This dataset was used for the identification of positive selection in this genetic region and the same sampling procedure was employed as in the source publication [14].

To infer positive selection, we measured the Extended Haplotype Homozygosity (EHH), defined as the probability that any two randomly chosen chromosomes carrying the haplotype of interest are identical by descent from the core region to a distance *x*. We also performed the Relative EHH (REHH) test, which compares the factor by which EHH decays on the tested haplotype to the decay on all other haplotypes combined. We selected one SNP at a time as the core marker and then computed the REHH for each haplotype and compared values at increasing distances from these markers. These analyses were performed with Sweep [18] and haplotype phases were estimated with BEAGLE 3.3.2 [19]. The integrated haplotype score (*iHS*) [20], which is a measure of the amount of EHH at a given SNP along the ancestral allele relative to the derived allele, and its significance was calculated using the ‘rehh’ package [21] in R.

A negative *iHS* indicates greater homozygosity surrounding the derived allele and a positive *iHS* indicates greater homozygosity surrounding the ancestral allele. Both indicate positive selection and its significance was indirectly used to assess EHH tests significance.

### Allele Frequency Differential in Simulated and Genome-Wide Empirical Data

To test how the observed patterns of *ACTN3* genetic diversity are correlated with neutral evolution and how it compares with genome-wide allelic empirical distribution, we simulated genetic data for 1000 SNPs with ms [22] under a wide range of demographic scenarios that mimic the prehistoric settlement of the American continent and calculated the frequency differential between East Asians and Amerindians for over 660 thousand SNPs in the HGDP database [12]. We chose America since this is where the highest frequencies of the *577X* allele were observed [8].

For the simulation approach, we employed a similar methodology to that developed by Schroeder et al. [23], in which we analyzed a dataset simulated under strict neutrality to determine how often we observed an allele with a similar distribution to that described for the polymorphism under investigation. The polymorphism from our study, the rs1815739, presents an allele with high frequency in America and an average allele frequency differential of 0.29 in comparison to their ancestors, the East Asians (see Table 1 in the Results section).

**Table 1:** Frequencies of the rs1815739 derived allele in autochthonous populations pooled into seven geographical groups.

Geographical group	Sample sizes (2n)	*577X* allele frequencies
Africa	794	0.093
Middle East	356	0.392
Europe	445	0.443
Central and South Asia	199	0.502
East Asia	581	0.477
Oceania	35	0.495
Americas	394	0.764

**Fig 1:**
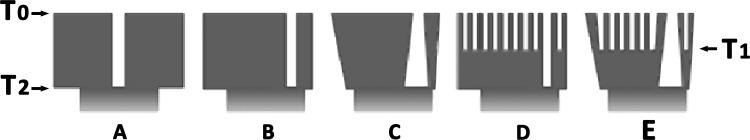
Demographic models used for the coalescent simulations: A—population split at T_2_ with two derived populations of the same size; B—population split at T_2_ with two derived populations with ratio between their effective population sizes (N_EF_) equal to 0.15; C—population split at T_2_ with two derived populations with the ratio between their N_EF_s equal to 0.06 at T_2_ and 0.15 presently (T_0_); D—same as model B, but with population substructure arising at T_1_ in both derived populations; E—same as model C, but with population substructure arising at T_1_ in both derived populations. The number of demes depicted does not correspond to the actual simulations. For further details see methods section.

Five scenarios (Fig. 1) were modeled for the split of Native Americans from Asians, as follows:

- Model A considers two derived populations with the same size;
- Model B adds a bottleneck to the previous model, such that the ratio between the effective population sizes (N_EF_) of the derived populations is 0.15;
- In Model C, this ratio is 0.06 and both populations undergo exponential growth so that the ratio between N_EF_s of the derived populations is 0.15 in the present (T_0_);
- Model D is the same as B, but with population substructure in both derived populations;
- Model E is the same as C, but with population substructure in both derived populations.

Population samples and deme sizes were defined based on the empirical data available for the rs1815739 polymorphism. We excluded those populations with less than 4 available genotypes, and only considered Asians as the derived non-Native American population, since populations from this region are the most genetically related to present day Amerindians. That yielded 25 demes for the simulated population that mimicked Asia and 18 for the simulated populations that mimicked the America continent.

Three different splitting times (T_2_) between Asia and the Americas were modeled for each scenario as follows: 1,001, 740, and 500 generations before the present. Current Asian N_EF_ was assumed to be n = 9,000 and the ancestral N_EF_ was equal to that estimated for Asia during the split, which may vary according to the model as explained before.

For models D and E, structure was set to emerge at T_1_, which assumed different values in different runs (295, 195, or 75 generations ago). Migration between the subpopulations was introduced to the simulations according to stepping-stone and island migration models separately. Under the island model, in each generation, 5% of each subpopulation consisted of a random selection of migrants from all other subpopulations. For the stepping-stone model, the same percentage was comprised of migrants from two other subpopulations. We also considered post-Beringia circumarctic gene flow [24]; according to this proposal, gene flow would also occur between one Asian and one American subpopulation at the rate of 0.0022, and they would not exchange migrants with the other subpopulations from their respective continents.

This procedure replicated Schroeder et al.’s strategy [23]. The ms command-lines employed were based on those kindly provided by the aforementioned authors and are available as the S1 File online. Modifications were performed to accommodate differences between sampling schemes and genetic systems in the two studies.

In addition to the simulations, we evaluated how the allele frequency differential observed for the rs1815739 compares to the empirical distribution of allele frequency differentials between East Asians and Amerindians using genome-wide data for over 660,000 SNPs from HGDP [12]. This empirical distribution’s summary-statistics were calculated with the command-line version of Arlequin ver 3.5.1.3 [17] and in-house built bash scripts. Measures of central tendency and the cumulative distribution for this measures were computed with R [25].

### ACTN3*577X Variation in Archaic Humans

We checked the genotypes for rs1815739 in the extinct humans [26, 27] and archaic *Homo sapiens* individuals from Mal’ta in south-central Siberia [28] and the Clovis burial site in North America [29]. For accessing this information from VCF or BAM files, we used VariantAnnotation [30] and Samtools [31] packages.

## Results

Table 1 shows the *577X* allele frequencies in the analyzed populations pooled into seven major geographical regions. Mozabite was excluded from Africa due its location and possible influence of Middle Eastern gene flow. S1 Table presents the *577X* allele distribution for each population before pooling. Using population frequency data from African samples, the allele age for ACTN3*577X was estimated to be 61,373 ± 27,783 YBP (∼2,455 ± 1,110 generations).

**Fig 2:**
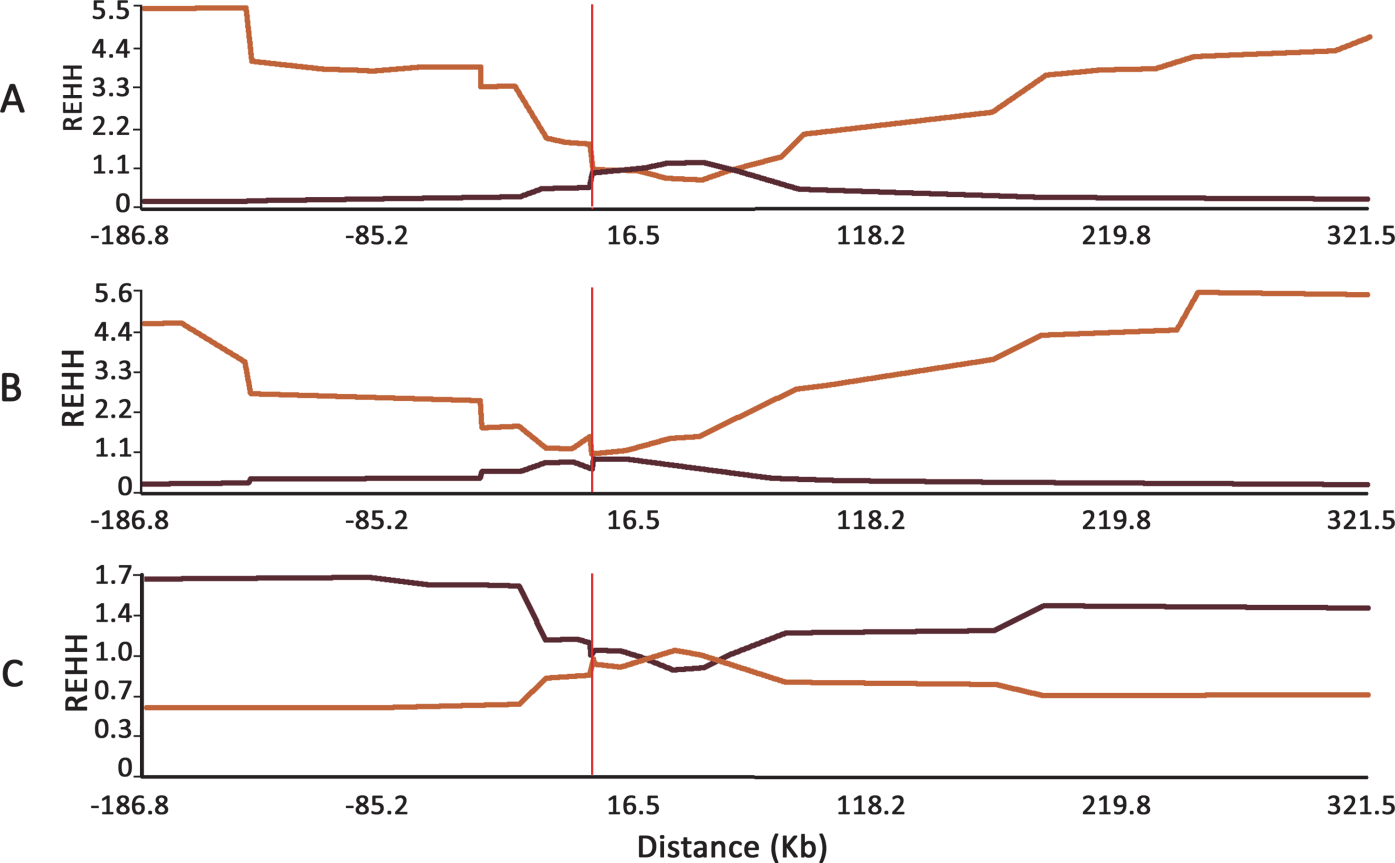
Relative extended haplotype homozygosity (REHH) for 30 SNPs located at a distance described in the X-axis from *ACTN3* rs1815739 spanning circa 510 kb. The orange line represents the homozygosity of the flanking SNPs relative to rs1815739 for haplotypes carrying the derived *577X* allele, while the red line represents the same for haplotypes carrying the ancestral allele (*i.e*. for Asia, REHH indicates that extended haplotype homozygosity-EHH, considering *577X*-bearing chromosomes, is about 5.5 times greater than the average EHH of the others bearing *R577*). A: Asian; B: European; and C: American data.

The data from Asian and European populations (Fig. 2—A and B respectively) showed that the REHH values of the *577X* allele-bearing chromosomes are higher than the REHH of the ancestral allele-bearing chromosomes on both sides of rs1815739 (REHH = 5.5 and 4.4 at 187 kb of distance on the 5’ side; and REHH = 5.3 and 5.6 at 322 kb on the 3’ region, for Asian and Europeans, respectively). In other words, for Asia, the REHH indicates that for *577X*-bearing chromosomes extended haplotype homozygosity-EHH is about 5.5 times greater than the average EHH of chromosomes bearing *R577*. Native Americans presented lower extension values (REHH = 1.7 at 187 kb on the 5’ side, and REHH = 0.7 at 322 kb on the 3’ region; Fig. 2—C).

The integrated haplotype score (*iHS*) showed greater homozygosity surrounding the derived allele, indicating positive selection for the *577X* in Asians (*p* = 0.000293). On the other hand, the p values for Native Americans were not statistically significant.

Considering the neutral demographic simulation tests, on average 6.1% of SNPs reproduced the pattern of the rs1815739 polymorphism observed in the Americas. Models A, B, C, D, and E respectively present 0.8, 1.8, 10.5, 6.9, and 6.4% of SNPs with an allelic distribution similar to that of the rs1815739, that is, a frequency differential of at least 0.29 between the populations. In general, this percentage was higher with increasingly more complex and realistic models. The inclusion of a bottleneck in the simulation representing the Native American population, for instance, was the factor that most contributed to the emergence of high-frequency alleles in America. This provides evidence for the role of this evolutionary force in generating the observed allelic distribution pattern. Different migration models (i.e. island and stepping-stone) did not affect the emergence of such intercontinental differentiation for this allele. Meanwhile circumarctic migration, when introduced, slightly lowered the proportion of simulations presenting the rs1815739 polymorphism pattern. In other words, despite the low proportion of loci mimicking the ACTN3*R577X distribution, the simulated scenarios could capture some features of demographic dynamics during the peopling of the New World, revealing specific features’ roles in generating such a peculiar allelic distribution.

Twelve percent of the SNPs in HGDP database present a larger allele frequency differential between East Asians and Native Americans than that observed for the rs1815739 (Minimum observed allele frequency differential = 0.000; First quartile = 0.031; Median = 0.099; Third quartile = 0.199; Maximum = 0.896).

All archaic humans were homozygous for the ancestral allele C allele in rs1815739, but the ancient Native American from the Clovis burial site (homozygous for derived allele T), which dates from 13,000 to 12,600 calendar years BP [29].

## Discussion

Human populations underwent many different bottlenecks as they expanded their range across the planet [32, 33]. These bottlenecks affect genetic diversity such that one allele may become fixed in some groups while reaching intermediate or very low frequencies in others. In fact, high interpopulation diversity (measured by statistics such as *Fst*) due solely to neutrality is expected in a large proportion of loci in the human genome [34]. This phenomenon is particularly striking in Native Americans when compared to other major human geographic groups [35]; on the other hand, the different selective pressures that humans encountered during the colonization of the world may also have caused allele frequency heterogeneity between populations, since some alleles may be more advantageous than others in specific environments [36]. Dissecting the effects of drift from those of natural selection may be a hard task in some cases. In this study, we showed the importance of both processes to explain the pattern of *ACTN3* allele distribution in the major human continental evolutionary groups, especially for Native Americans.

Some studies have suggested that *ACTN3* may be evolving under natural selection [6, 7, 9] and another has demonstrated it empirically [5]. Moreover, positive selection upon *ACTN3* in certain human groups can also be identified with the HGDP Selection Browser [37]. Our study also favors the hypothesis of an adaptive evolutionary history for rs1815739 at *ACTN3*, although random drift may also be important in some regions of the world.

The ACTN3*577X derived allele frequency shows a general trend of increase with distance from Africa, reaching its highest frequencies in the Americas (Table 1). Despite it, the corresponding adaptive sweep could not be confirmed in the New World, since REHH values for Native Americans were about five times lower than those found for the haplotypes carrying the derived *577X* allele in Asia and Europe, in addition to the observed non-significant values in *iHS* tests. A few hypotheses for the high fitness of carriers of the *577X* allele in Eurasia can be suggested, such as muscle activity advantages in endurance running. In scenarios such as those proposed by Bramble and Lieberman [6], this corresponds to ease of access to carcasses and the emergence of new strategies for acquiring food, such as persistence hunting. In this regard, the present estimate of the allele’s emergence at 61.4 thousand years ago, although within a large confidence interval, is placed in the range calculated for the first human migrations out of Africa (45.0 to 87.5 thousand years ago [38]), providing further support for the hypothesis that it may have presented selective advantages during *Homo sapiens*’ dispersal from Africa. The presence of this allele in homozygosis in a archaic individual from one of those which are believed to be the oldest Native American cultures [29] shows that this allele could have had a high frequency in the Native American ancestral population in Asia and therefore have remained segregating in the Americas even after a strong bottleneck associated with the peopling of Beringia a few thousand years before [32].

In accordance with the lack of EHH test evidence for *ACTN3* positive selection in the Americas, the neutral demographic simulations also suggest alleles may have raised in frequency on this continent due to drift effects, without the need to invoke adaptive processes. Scenario C, which includes a population bottleneck and exponential growth (see Methods section), yielded the highest number of loci with allele frequency differential similar to that observed for the rs1815739. This number is the most similar to that observed for genome-wide SNP data, but is still lower than that, what could be due to the lack of certain details of the demographic dynamics of the peopling of the Americas from the devised models or, on the other hand, the effect of natural selection on part of the human genome. Alternately, this may indicate that the emergence of alleles in neutral loci with high frequency differentials between Native Americans and their Asian ancestors is actually very rare, even in face of population bottlenecks and reduced effective size, and that selective forces may be in fact acting upon our genome-wide diversity (represented by HGDP SNP database).

Our study neither indicates that this allele presents selective advantage, nor that it clearly evolves due solely to random drift. Rather, it suggests a combination of both factors, with possible changes in selective pressure over time and space, and the loss or fixation of the beneficial allele in certain groups due to drift.

Native American population structures can distort positive selection signals and make them hard to identify. Works on *PAX9* [39], *ABCA1* [40], and *KIR* [41] found similar results to those reported here. In these studies, no signal of positive selection was found in the Americas (or specifically in South America for *ABCA1*), despite the evidence for non-neutrality in populations from other regions. As more realistic simulations and robust selection inference methods are developed, a clearer evolutionary scenario for such genes in the America may be proposed.

The hypothesis of a recent high increase in frequency of the rs1815739 derived allele due to selection in the Americas is appealing. An allele that could enhance human dispersal would likely be advantageous during the settlement of the New World, especially since it happened over a relatively short time [42]. Moreover, the involved populations certainly have used persistence hunting and this way of living could act as a selective pressure favoring the derived allele. In spite of the doubts about the exact role of this allele in enhancing human fitness [3], our study does not exclude the view that the X allele may enhance human capability for enduring exercising and related practices and may have well presented a selective advantage for the spreading of modern humans all over the world.

## Supporting Information

S1 Table Frequency of the rs1815739 derived allele in autochthonous populations worldwide. (DOCX)

S1 File Command lines for simulating the diversity of 1000 neutral SNPs under different scenarios of settlement of the New World. (SH)
