# Supplementary material for: Differing Evolutionary Histories of the ACTN3*R577X Polymorphism among the Major Human Geographic Groups
Source: PLoS One. 2015 Feb 23;10(2):e0115449. doi: 10.1371/journal.pone.0115449 (PMC4338210; doi:10.1371/journal.pone.0115449)
Supplement: S1 Table — (DOCX) [file pone.0115449.s001.docx]

**Table S1:** Frequency of the rs1815739 derived allele in autochthonous populations worldwide

|  | **Population** | **Sample size**  **(2n)** | **577X**  **allele frequency** | **Reference** |
| --- | --- | --- | --- | --- |
| **Africa** | Bantu in Kenya | 12 | 0.08 | ^2^ |
|  | Bantu in South Africa | 38 | 0.11 | ^6^ |
|  | Biaka Pygmies | 30 | 0.13 | ^2^ |
|  | Gui Ghana Kgal | 14 | 0 | ^6^ |
|  | Herero | 16 | 0.12 | ^6^ |
|  | Juhoansi | 34 | 0 | ^6^ |
|  | Karretjie | 24 | 0 | ^6^ |
|  | Khomani | 34 | 0.06 | ^6^ |
|  | Khwe | 34 | 0.03 | ^6^ |
|  | Luhya | 97 | 0.08 | ^3^ |
|  | Luhya | 90 | 0.07 | ^1^ |
|  | Maasai | 143 | 0.19 | ^1^ |
|  | Mandenka | 24 | 0.17 | ^2^ |
|  | Mbuti Pygmies | 15 | 0.03 | ^2^ |
|  | Mozabite | 30 | 0.48 | ^2^ |
|  | Nama | 14 | 0.07 | ^6^ |
|  | San | 6 | 0 | ^2^ |
|  | Xun | 26 | 0.08 | ^6^ |
|  | Yoruba | 113 | 0.08 | ^1^ |
| **Middle East** | Bedouin | 48 | 0.42 | ^2^ |
|  | Druze | 47 | 0.29 | ^2^ |
|  | Palestinian | 51 | 0.42 | ^2^ |
|  | Iran | 210 | 0.44 | ^4^ |
| **Europe** | Adygei | 17 | 0.56 | ^2^ |
|  | British | 89 | 0.49 | ^3^ |
|  | Finnish | 93 | 0.35 | ^3^ |
|  | French | 29 | 0.41 | ^2^ |
|  | French Basque | 24 | 0.46 | ^2^ |
|  | Spanish | 14 | 0.39 | ^3^ |
|  | North Italian | 12 | 0.42 | ^2^ |
|  | Orcadian | 16 | 0.59 | ^2^ |
|  | Russian | 25 | 0.3 | ^2^ |
|  | Sardinian | 28 | 0.48 | ^2^ |
|  | Toscan | 98 | 0.42 | ^3^ |
| **Central and South Asia** | Balochi | 25 | 0.46 | ^2^ |
|  | Brahui | 25 | 0.32 | ^2^ |
|  | Burusho | 25 | 0.52 | ^2^ |
|  | Hazara | 17 | 0.38 | ^2^ |
|  | Kalash | 25 | 0.86 | ^2^ |
|  | Makrani | 25 | 0.46 | ^2^ |
|  | Pathan | 22 | 0.48 | ^2^ |
|  | Sindhi | 25 | 0.54 | ^2^ |
|  | Uygur | 10 | 0.50 | ^2^ |
| **East Asia** | Altaian | 3 | 0.67 | ^5^ |
|  | Buryat | 9 | 0.61 | ^5^ |
|  | Cambodians | 11 | 0.45 | ^2^ |
|  | Chukchi | 30 | 0.3 | ^5^ |
|  | Dai | 10 | 0.4 | ^2^ |
|  | Daur | 9 | 0.61 | ^2^ |
|  | Dolgan | 4 | 0.62 | ^5^ |
|  | Evenki | 10 | 0.4 | ^5^ |
|  | Han Chinese | 97 | 0.41 | ^3^ |
|  | Hezhen | 8 | 0.44 | ^2^ |
|  | Japanese | 89 | 0.49 | ^3^ |
|  | Ket | 1 | 1 | ^5^ |
|  | Khanty | 5 | 0.6 | ^5^ |
|  | Koryak | 10 | 0.3 | ^5^ |
|  | Lahu | 10 | 0.35 | ^2^ |
|  | Miaozu | 10 | 0.4 | ^2^ |
|  | Mongolia | 10 | 0.5 | ^2^ |
|  | Naukan | 16 | 0.41 | ^5^ |
|  | Naxi | 9 | 0.56 | ^2^ |
|  | Nganasan | 20 | 0.43 | ^5^ |
|  | Oroqen | 10 | 0.55 | ^2^ |
|  | Selkup | 4 | 0.5 | ^5^ |
|  | She | 10 | 0.35 | ^2^ |
|  | Southern Han Chinese | 100 | 0.38 | ^3^ |
|  | Tu | 10 | 0.5 | ^2^ |
|  | Tujia | 10 | 0.35 | ^2^ |
|  | Tuvinians | 9 | 0.39 | ^5^ |
|  | Xibo | 9 | 0.33 | ^2^ |
|  | Yakut | 25 | 0.48 | ^2^ |
|  | Yizu | 10 | 0.5 | ^2^ |
|  | Yukaghir | 13 | 0.5 | ^5^ |
| **Oceania** | Melanesian | 18 | 0.64 | ^2^ |
|  | Papuan | 17 | 0.35 | ^2^ |
| **Americas** | Algonquin | 2 | 1 | ^5^ |
|  | Arara | 1 | 0 | ^5^ |
|  | Arhuaco | 1 | 1 | ^5^ |
|  | Aymara | 19 | 0.89 | ^5^ |
|  | Bribri | 4 | 0.87 | ^5^ |
|  | Cabecar | 30 | 0.6 | ^5^ |
|  | Chané | 2 | 1 | ^5^ |
|  | Chilote | 4 | 0.75 | ^5^ |
|  | Chipewyan | 7 | 1 | ^5^ |
|  | Chono | 1 | 1 | ^5^ |
|  | Colombian Amerindians | 13 | 0.81 | ^2^ |
|  | Cree | 2 | 1 | ^5^ |
|  | Diaguita | 3 | 1 | ^5^ |
|  | Inuit | 4 | 0.25 | ^5^ |
|  | Embera | 5 | 0.8 | ^5^ |
|  | Guahibo | 6 | 0.67 | ^5^ |
|  | Guarani | 5 | 0.7 | ^5^ |
|  | Guaymi | 5 | 0.8 | ^5^ |
|  | Huilliche | 3 | 0.83 | ^5^ |
|  | Inga | 6 | 1 | ^5^ |
|  | Jamamadi | 1 | 0 | ^5^ |
|  | Kaingang | 1 | 0 | ^5^ |
|  | Kaqchikel | 10 | 0.7 | ^5^ |
|  | Karitiana | 24 | 0.9 | ^2^ |
|  | Kogi | 3 | 0.5 | ^5^ |
|  | Maleku | 2 | 0.75 | ^5^ |
|  | Maya | 25 | 0.88 | ^2^ |
|  | Mixe | 17 | 0.91 | ^5^ |
|  | Mixtec | 5 | 0.75 | ^5^ |
|  | Ojibwa | 2 | 1 | ^5^ |
|  | Palikur | 3 | 1 | ^5^ |
|  | Parakanã | 1 | 0.5 | ^5^ |
|  | Pima | 25 | 0.88 | ^2^ |
|  | Purepecha | 1 | 1 | ^5^ |
|  | Quechua | 38 | 0.89 | ^5^ |
|  | Surui | 21 | 0.48 | ^2^ |
|  | Tepehuano | 23 | 0.76 | ^5^ |
|  | Teribe | 3 | 1 | ^5^ |
|  | Ticuna | 6 | 0.5 | ^5^ |
|  | Toba | 4 | 0.62 | ^5^ |
|  | Waunana | 3 | 1 | ^5^ |
|  | Wayuu | 9 | 0.72 | ^5^ |
|  | Wichi | 5 | 0.8 | ^5^ |
|  | Yaghan | 2 | 1 | ^5^ |
|  | Zapotec | 39 | 0.86 | ^5^ |

^1^International HapMap Consortium (2003); ^2^Li et al. (2008); ^3^1000 Genomes Project Consortium et al. (2012); ^4^Fattahi and Najmabadi (2012); ^5^Reich et al. (2012); ^6^Schlebusch et al. (2012).

1. International HapMap Consortium: The International HapMap Project. *Nature* 2003; **426**: 789-96.

2. Li JZ, Absher DM, Tang H et al.*:* Worldwide human relationships inferred from genome-wide patterns of variation*. Science* 2008; **319**: 1100-4.

3. 1000 Genomes Project Consortium, Abecasis GR, Auton A et al.: An integrated map of genetic variation from 1,092 human genomes. *Nature* 2012; **491**: 56-65.

4. Fattahi Z, Najmabadi H: Prevalence of ACTN3 (the athlete gene) R577X polymorphism in Iranian population. *Iran Red Crescent Med J* 2012; **14**: 617-22.

5. Reich D, Patterson N, Campbell D et al.*:* Reconstructing Native American population history. *Nature* 2012; **488**: 370-4.

6. Schlebusch CM, Skoglund P, Sjödin P et al.*:* Genomic variation in seven Khoe-San groups reveals adaptation and complex African history. *Science* 2012; **338**: 374-9.
